# CamMedNP: Building the Cameroonian 3D structural natural products database for virtual screening

**DOI:** 10.1186/1472-6882-13-88

**Published:** 2013-04-16

**Authors:** Fidele Ntie-Kang, James A Mbah, Luc Meva’a Mbaze, Lydia L Lifongo, Michael Scharfe, Joelle Ngo Hanna, Fidelis Cho-Ngwa, Pascal Amoa Onguéné, Luc C Owono Owono, Eugene Megnassan, Wolfgang Sippl, Simon MN Efange

**Affiliations:** 1 Department of Pharmaceutical Sciences, Martin-Luther University of Halle-Wittenberg, Wolfgang-Langenbeck Str. 4, Saale, Halle, 06120, Germany; 2 CEPAMOQ, Faculty of Science, University of Douala, P.O. Box 8580, Douala, Cameroon; 3 Department of Chemistry, Faculty of Science, University of Buea, P. O. Box 63, Buea, Cameroon; 4 Department of Chemistry, Faculty of Science, University of Douala, P. O. Box 24157, Douala, Cameroon; 5 Department of Biochemistry and Molecular Biology, Faculty of Science, University of Buea, P. O. Box 63, Buea, Cameroon; 6 Laboratory for Simulations and Biomolecular Physics, Ecole Normale Supérieure, University of Yaoundé I, P.O. Box 47, Yaoundé, Cameroon; 7 Laboratory of Fundamental and Applied Physics, University of Abobo-Adjame, Abidjan 02, Cote d’Ivoire, BP, 801, Africa

**Keywords:** 3D structures, Database collection, Natural products, Medicinal plants, Virtual screening

## Abstract

**Background:**

Computer-aided drug design (CADD) often involves virtual screening (VS) of large compound datasets and the availability of such is vital for drug discovery protocols. We present CamMedNP - a new database beginning with more than 2,500 compounds of natural origin, along with some of their derivatives which were obtained through hemisynthesis. These are pure compounds which have been previously isolated and characterized using modern spectroscopic methods and published by several research teams spread across Cameroon.

**Description:**

In the present study, 224 distinct medicinal plant species belonging to 55 plant families from the Cameroonian flora have been considered. About 80 % of these have been previously published and/or referenced in internationally recognized journals. For each compound, the optimized 3D structure, drug-like properties, plant source, collection site and currently known biological activities are given, as well as literature references. We have evaluated the “drug-likeness” of this database using Lipinski’s “Rule of Five”. A diversity analysis has been carried out in comparison with the ChemBridge diverse database.

**Conclusion:**

CamMedNP could be highly useful for database screening and natural product lead generation programs.

## Background

For more than 4 millennia, plants have been used as a source of medication. According to the World Health Organization (WHO), phytomedicine is a part of health care systems around the world [1], and its importance is underscored by the fact that by 1990 about 80% of drugs were either natural products (NPs) or analogues inspired by them [2-4]. Moreover, large proportions of natural products are biologically active and have favourable ADME/T (absorption, distribution, metabolism, excretion, and toxicology) properties, despite the fact that they often do not satisfy proposed “drug-likeness” criteria [5]. Thus, modern drug discovery programs often resort to natural sources to guide the careful design of “drug-like” leads from suitable scaffolds, often by synthetic modifications of the latter [6,7]. Nowadays, employing computer-aided drug design (CADD) methods, which often incorporate the virtual screening (VS) of large compound databases against validated drug targets followed by the careful selection of virtual hit compounds to be screened by biological assays, has become a very important part of the drug discovery process. This strategy considerably narrows down the number of compounds that undergo biological screening and hence drastically cuts down the cost of discovery of a drug [7-10]. The adoption of this drug discovery strategy has therefore necessitated the development of databases of virtual compounds. In addition to the increasing number of commercial natural compound suppliers [11], the past decade has seen the development and publication of a number of NP compound databases: The SuperNatural database [12]; The Chinese traditional medicinal herbs database [13]; Marine natural products databases [14,15]; The NAPROC-13 database [16]; a database for the predicted pharmacophoric features of medicinal compounds isolated from medicinal plants in India [17,18]; and the PHARM database, based on Thai medicinal plants [19].

The fact that the African flora, and the Congo Basin in particular, holds enormous potential as a source of drugs for its poverty-stricken populations and the world at large cannot be overemphasized [20-22]. Located in the Congo Basin, Cameroon has a rich rain forest and most of her rural populations have depended on medicinal plants for the treatment of a number of tropical diseases until now [21,22]. Interest in these plants led to the creation of the Department of Organic Chemistry at the University of Yaoundé (now Yaoundé I, UY), whose NP research groups have been thereafter actively involved in the isolation and characterization of active principles from medicinal plants that could serve as drug leads. Moreover the research teams born in UY have served as the nursery for the training of more than 90% of the current leaders of the various NP research groups spread throughout the country. For more than four decades, Cameroonian research groups have been actively involved in the extraction, purification and characterization of biologically active compounds from medicinal plants. The result has been the steady increase in the volume of scientific publications annually. It must, however, be noted that only the most promising compounds are usually published in internationally recognized peer reviewed journals. The seemingly uninteresting ones are only reported in local journals as well as in MSc and PhD theses. The locally published and unpublished data, nevertheless, constitute an enormous wealth of knowledge that has remained unavailable to the wider scientific community. To the best of our knowledge, a searchable 3D compound database of pure compounds from Cameroonian medicinal plants has not been previously reported. Even though the chemical structures of about 80% of the compounds in CamMedNP are published in journal articles, the presence of 3D structures makes the present database valuable for molecular modelling groups carrying out VS and CADD. Moreover, little effort has been made locally to develop the expertise (in areas such as medicinal chemistry), which is required to mount credible drug development efforts [23]. It is therefore of value to present a comprehensive data review, based on the published and unpublished results of the various research groups. The goal has been to prepare a database containing 3D structures as well as the physico-chemical properties, geographical distribution of the plant species and the known biological activities of these compounds. In this paper, we present CamMedNP, a new database of 3D chemical structures, available in several file formats (.mdb, .ldb, .mol2, .sdf), which are readable using several drug discovery software tools. Thus, CamMedNP could be used by research groups involved in CADD to carry out protein-ligand docking, pharmacophore mining, substructure searching and VS against validated drug targets. Since these plants have been used traditionally in the treatment of several medical disorders, the aim of VS would be to identify suitable compound scaffolds which could be subjected to further investigation in the search for lead compounds for the treatment of these and other diseases. An assessment of the “drug-likeness” of the CamMedNP database in comparison with the Dictionary of Natural Products (DNP) is also reported here, as well as a diversity analysis, in comparison with the ChemBridge diverse database.

## Construction and content

### Data sources

The plant sources, geographical collection sites, chemical structures of pure compounds as well as their spectroscopic data, were retrieved from literature sources comprising of 25 PhD and Habilitation theses from the libraries of the University of Yaoundé I (UY), University of Douala (UD), University of Dschang (UDs) and the University of Buea (UB), all in Cameroon. International peer reviewed journal sources include 48 journals, with references ranging from 1982 to 2012. This constitutes a total of 364 journal references, as well as 4 unpublished conference presentations (from personal communication with the authors). The full list of journals consulted is given in the supplementary material (Additional file 1).

### Generation of 3D Models, Optimization and Calculation of Molecular Descriptors

Based on the known chemical structures of the NPs, all 3D molecular structures were generated using the graphical user interface (GUI) of the MOE software [24] running on a Linux workstation with a 3.5GHz Intel Core2 Duo processor. The 3D structures were generated using the builder module of MOE and energy minimization was subsequently carried out using the MMFF94 force field [25] until a gradient of 0.01 kcal/mol was reached. The 3D structures of the compounds were then saved as .mol2 files subsequently included into a MOE database (.mdb) file and converted to other file formats (.sdf, .mol, .mol2 and .ldb), which are suitable for use in several virtual screening workflow protocols. The molar weight (MW), number of rotatable bonds (NRB), lipophilicity parameter (log *P*), number of hydrogen bond acceptors (HBA), number of hydrogen bond donors (HBD), number of Lipinski violations, total polar surface area (TPSA), number of nitrogens (NN), number of oxygens (NO), number of chiral centres (NCC), and number of rings (NR) were calculated using the molecular descriptor calculator included in the QuSAR module of the MOE package [24]. The ChemBridge Diverset database (48,651 compounds) was downloaded from the official ChemBridge webpage [26]. It is noteworthy that the provided 3D structures are those published in the literature, based on NMR and other spectroscopic techniques. Standard software programs which implement typical virtual screening workflows usually involve a preliminary treatment of input ligand structures by tautomer generation and correct protonation at physiological pH. It is the user’s responsibility to implement this preliminary step during the virtual screening protocol.

### Plant families and their chemo-taxonomic classification

**Figure 1:**
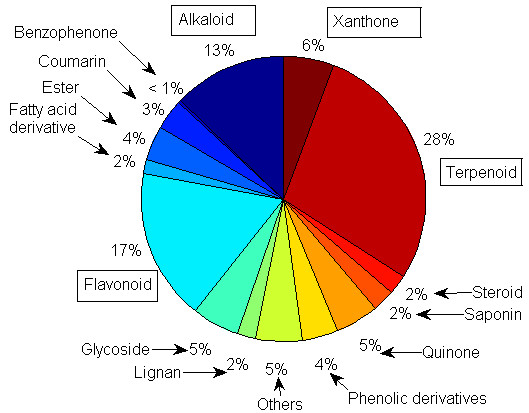
Pie chart showing the classification of compound types currently within the CamMedNP database.

**Figure 2:**
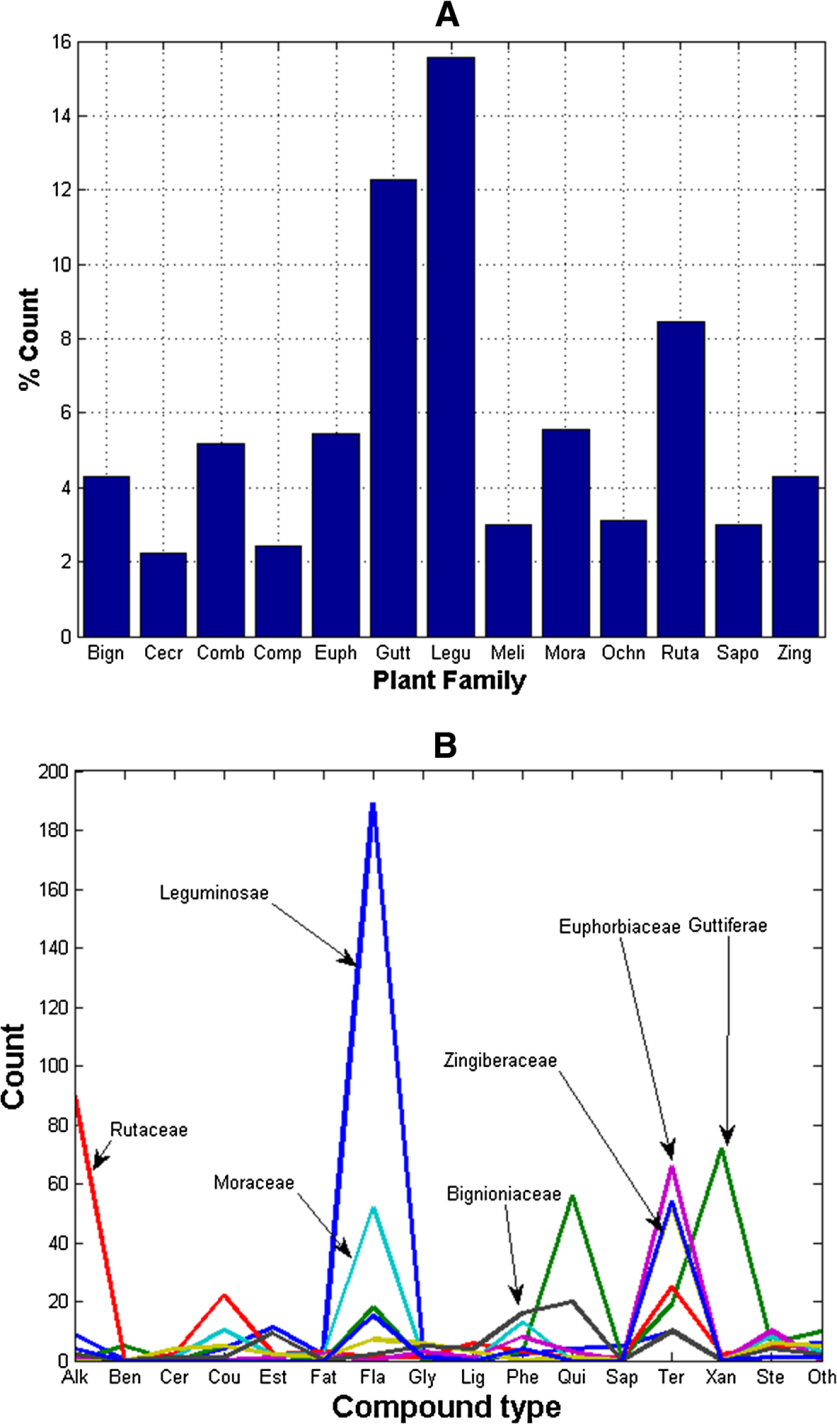
**Chemical composition by plant family of origin.** (**A**) Bar chart showing the % counts of compounds currently within the CamMedNP database, classified by plant family of origin. (**B**) Distribution of compound types within the most abundant plant families currently in CamMedNP. Each plant family is represented by the first 4 letters of its name (e.g., Bign = Bignioniaceae, …). Codes for compound types, represented in Figure 1, are given by the first 3 letters (e.g., Alk = Alkaloid, …).

Our initial collection gave 2,434 naturally occurring compounds, alongside 147 products of hemisynthesis and enzymatic biotransformations of the NPs. The NPs had been previously isolated from 224 plant species from 55 plant families. However, since the same compound can be isolated from several plant species, the removal of duplicates resulted in a total of 1,859 NPs and their derivatives, among which 33.8% have been derived from Cameroonian medicinal plants for the very first time. Duplicates were defined as compounds which have been reported from more than one species, more than one family or from the same species, but with different literature sources. Our analyses have shown that a plurality (solely based numbers, not relative concentrations) of the NPs currently included is made of terpenoids, constituting 28.4%. This is followed respectively by flavonoids (17.2%), alkaloids (12.8%), xanthones (5.7%), glycosides (5.4%) and quinones (5.0%), Figure 1. A classification of the molecules by plant family of origin showed that the most abundant molecules have been isolated from plants of the Leguminosae family (15.6%), followed by the Guttiferae (12.3%), Rutaceae (8.4%), Moraceae (5.5%), Euphorbiaceae (5.4%), Compositae (5.2%), Bignioniaceae (4.3%) and Zingiberaceae (4.3%) families respectively (Figure 2A). A chemo-taxonomic study of the compounds from the Cameroonian rainforest, currently included within this database reveals that plants of the Leguminosae family (*Crotalaria sp*., *Eriosema sp*., *Erythrina sp*., *Guibourtia sp*. and *Millettia sp*.) are particularly rich in flavonoids (constituting 75.6% of all compounds that have been isolated from this family), Figure 2B. On the other hand, it was observed that extracts from the Guttiferae family (*Allanblackia sp*., *Calophyllum sp*., *Endodesmia sp*., *Garcinia sp*., *Harungana sp*., *Hypericum sp*., *Pentadesma sp*., *Psorospermum sp*., *Symphonia sp*. and *Vismia sp*.) are mostly composed of xanthones (36.5%), and quinones (28.4%), while those from the Rutaceae family (*Afraegle sp*., *Araliopsis sp*., *Basalmocitrus sp*., *Clausena sp*., *Fagara sp*., *Oricia sp*., *Oriciopsis sp*., *Teclea sp*., *Vepris sp*., and *Zanthoxylum sp*.) are rich in alkaloids (53.3 %). Additionally, compounds isolated from plants of the Moraceae (*Dorstenia sp*., *Ficus sp*., *Morus sp*., and *Trilepisium sp*.) were mostly flavonoids (50.0%); meanwhile plants of the Euphorbiaceae family (*Alchornea sp*., *Croton sp*., *Discoglypremna sp*., *Drypetes sp*., *Fontainea sp*., *Macaranga sp*., *Maesobotrya sp*., *Neoboutonia sp*., and *Uapaca sp*.) are shown to be rich in terpenoids (69.5%). The same trend was seen among plants of the Cecropiaceae family (not shown in Figure 2B). It was also observed that plants from the Compositae family (*Chromoleana sp*., *Crassocephalum sp*., *Crepis sp*., *Echinops sp*., *Elephantopus sp*., *Helichrysum sp*., *Microglossa sp*., *Senecio sp*., and *Tithonia sp*.) were mostly rich in terpenoids (57.0%) while plants from the Bignioniaceae (*Kigelia sp*., *Newbouldia sp*., *Spathodea sp*. and *Stereospermum sp*.) and Zingiberaceae (*Aframomum sp*. and *Renealmia sp*.) families were respectively rich in quinones (26.3%) and terpenoids (67.5%). In Figure 2A, plant families with less than 2% isolated compounds have been left out of the discussion for the sake of clarity.

### Secondary metabolites isolated and their known biological activities

The reported biological activities of CamMedNP have also been included in our database. From our study, it was observed that even though the biological activities of 54.9% of the compounds have not been determined, the remaining compounds show a wide range of reported activities. Among the known biological activities are unspecific classifications like antimalarial, antileishmanial, antitubercular, antitrypanosomal, anti-HIV, antiinflamatory and analgesic, antioxidant, free radical scavenging, antiproliferative, cytotoxicity, erythrocyte susceptibility, spasmogenic, antidiabetic, herbicidal, hepatoprotective, cardiovascular, immunoinhibition, immunomodulatory, antisalmonellal, vasodilator, vasorelaxant and hypertensive effects and activity against *Onchocerca gutturosa*, while very specific descriptions like inhibition or modulation of known drug targets include: α-glucosidase inhibition, butyrylcholinesterase inhibition, urease inhibition, inhibition of phosphodiesterase I and xanthine oxidase, monoamine oxidase inhibition, prolyl endopeptidase and thrombin inhibition, antitumor, 11β-hydroxysteroid dehydrogenase inhibition, cholinesterase inhibition, prolyl endopeptidase I inhibition, inhibition of neuraminidases from *Clostridium perfringens* and *Vibrio cholerae*, snake venom phosphodiesterase I inhibition, inhibition of Phospholipase C, β-D-glucosidase, β-glucuronidase and α-D-Mannosidase inhibition, etc., cytotoxicity against *Mucor miehei* and *Artemia salina*, algicidal activity against *Chlorella fusca*, etc. against *Bacillus subtilis* ATCC 6633, activity against gram-positive *Bacillus megaterium*, etc., cytotoxic activity against the HT-29 and HCT 116 human colon cancer cell lines, against colorectal human cancer cells, against human promyelocytic leukemia (HL-60), human hepato-cellular carcinoma and against the human Caucasian prostate adenocarcinoma cell line PC-3, enhancement of cAMP-regulated chloride conductance of cells expressing CFTRΔF508, human neutrophil respiratory burst inhibition, and estrogenic activities. For the majority of cases, antiplasmodial activity was measured by inhibition of the chloroquine-resistant W2 *P. falciparum* strain with IC_50_ < 5 μM. Cytotoxicity measurements were carried out in potato disk tumor induction assay and some compounds showed interesting inhibitory properties against human DU-145 and hepatocarcinoma Hep G2 cells with >70% inhibition at 50 μg/mL. Anti-salmonellal assays were measured by minimum inhibitory concentration (MIC) and minimum bactericidal concentration (MBC) values of respectively in μg/mL against *Salmonella typhi*, *S. paratyphi A* and *S. paratyphi B*, and most compounds showed < MBC values of 100 μg/mL. Full details of the other assays could be obtained by consulting the cited references within the database.

## Utility and discussion

### Discussion of Lipinski’ “drug-likeness” criteria

**Figure 3:**
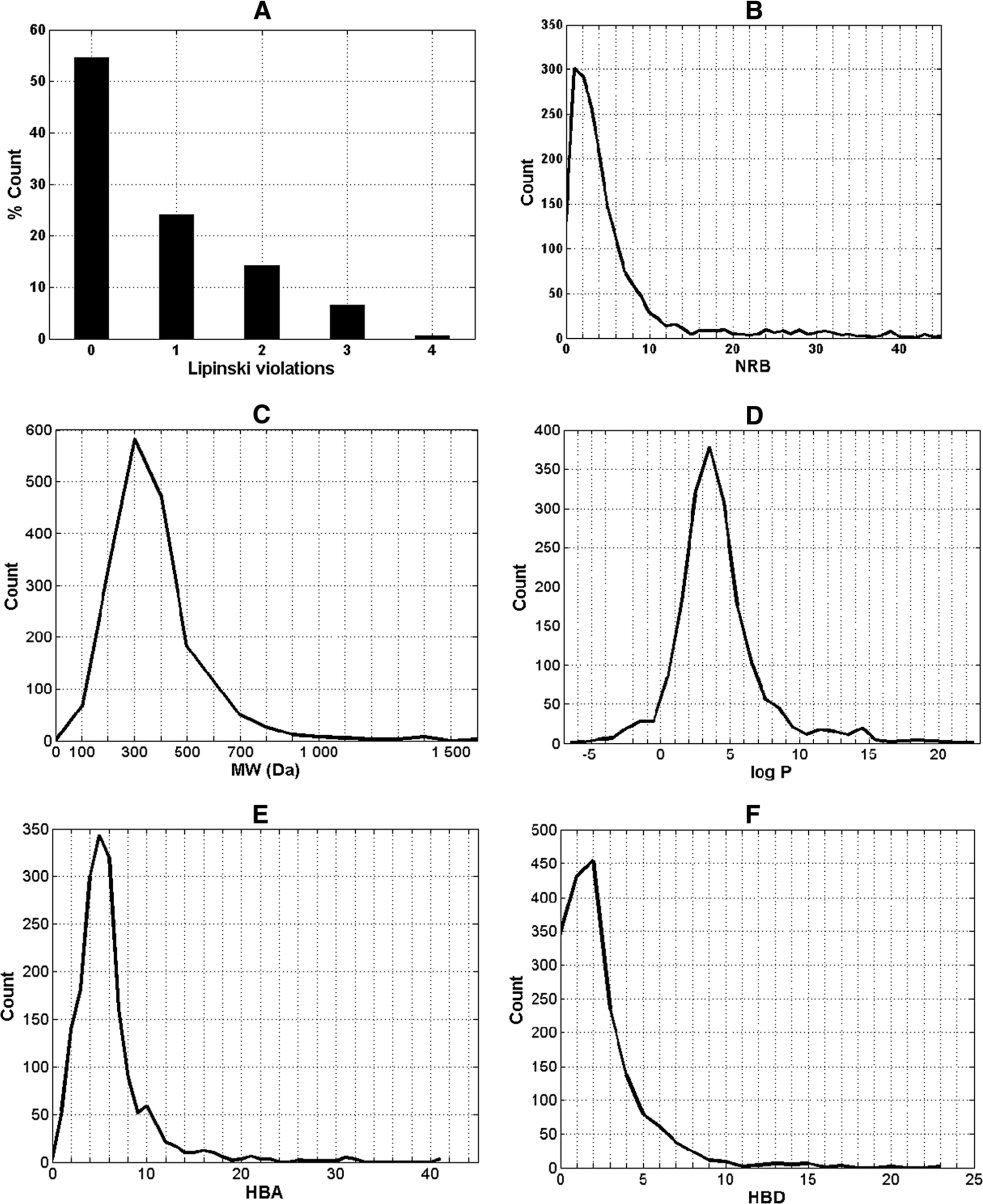
**Graph distribution of features that determine “drug-likeness”.** (**A**) Histogram of Lipinski violations as a percentage of the CamMedNP data set. (**B**, **C**, **D**, **E**, **F**) Distribution curves of the NRB, MW, log *P*, HBA and HBD respectively for the 1,859 compounds currently in CamMedNP. For subfigure **C**, the *x*-axis label is the lower limit of binned data, e.g. 0 is equivalent to 0 to 100.

**Figure 4:**
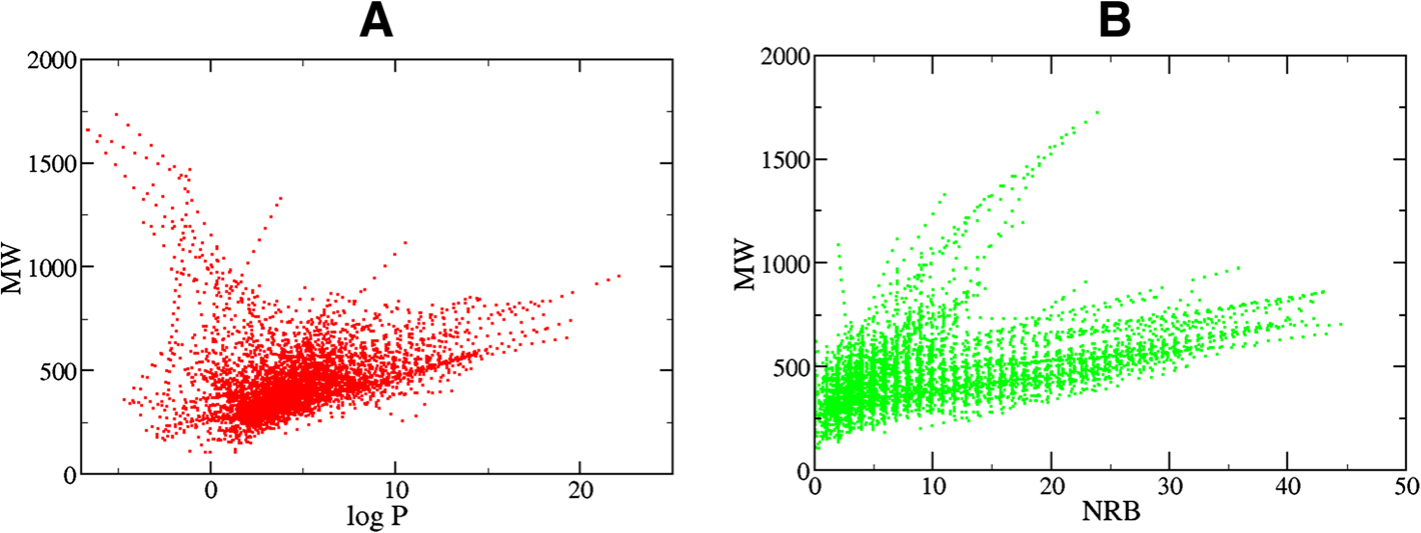
**Pairwise comparison of mutual relationships between molecular descriptors.** (**A**) The distribution of the calculated log *P* versus MW and (**B**) NRB versus MW.

Nowadays, the identification of lead compounds often involves the development of compound libraries with a high level of molecular diversity within the limits of significant “drug-like” properties. On these grounds, Lipinski’s criteria [27] have been used in the evaluation of the “drug-likeness” of the compounds within the CamMedNP database. The distributions of the compound molecular weights (MW), lipophilicity (log *P*), number of hydrogen bond acceptors (HBA), number of hydrogen bond donors (HBD) were calculated and used to compare the “drug-likeness” of CamMedNP with 126,140 compounds from the Dictionary of Natural Products (DNP) [28], previously analyzed, and retrieved from the literature [5]. It is noteworthy that natural products exhibit a wide range of flexibility, from rigid conformationally constrained molecules to very flexible compounds. Thus, the number of rotatable bonds (NRB) within the CamMedNP library was used as an additional criterion to test for the favourable drug metabolism and pharmacokinetics (DMPK) outcomes. It was observed that 54.6% of the compounds within CamMedNP showed no Lipinski violations and 78.7% showed < 2 violations (Figure 3A), while the peak of the distribution of the NRB was between 1 and 2 (Figure 3B). Moreover, the analysis of the distributions of MW showed a peak value between 301 and 400 Da (Figure 3C), with a curve similar to those previously reported for other “drug-like” NP libraries in the literature [5,29] and about 22% of MW > 500 Da. The distribution of the log *P* values showed a Gaussian shaped curve with a peak centred at 3.5 log *P* units (Figure 3D). However, some of the compounds had exceptionally large log *P* values, which went up to > 22 units. This may be explained by the fact that the training database/algorithm used to calculate log *P* may not suit the types and combinations of functional groups found in natural products [5]. It should however be noted that, inspite of this limitation, 73.1% of the compounds from CamMedNP had log *P* values < 5 units. The peaks of the HBA and HBD were respectively at 5 acceptors and 2 donors and both curves fell off rapidly to maximum numbers of 41 and 23 respectively (Figures 3E-F). It was also noted that only 8.6% of the compounds in CamMedNP had HBA > 10 and only about 9.4% had HBD > 5. Additionally, the pairwise comparison displaying the mutual relationship between the molecular weight versus the calculated log *P* and the number of rotatable bonds are specified in Figures 4A and 4B, respectively. The plots show that the regions with the highest population densities fall within the “Lipinski region of interest” (MW < 500, -2 < log *P* < 5), and for which NRB < 5.

### Comparison with the dictionary of natural products

**Figure 5:**
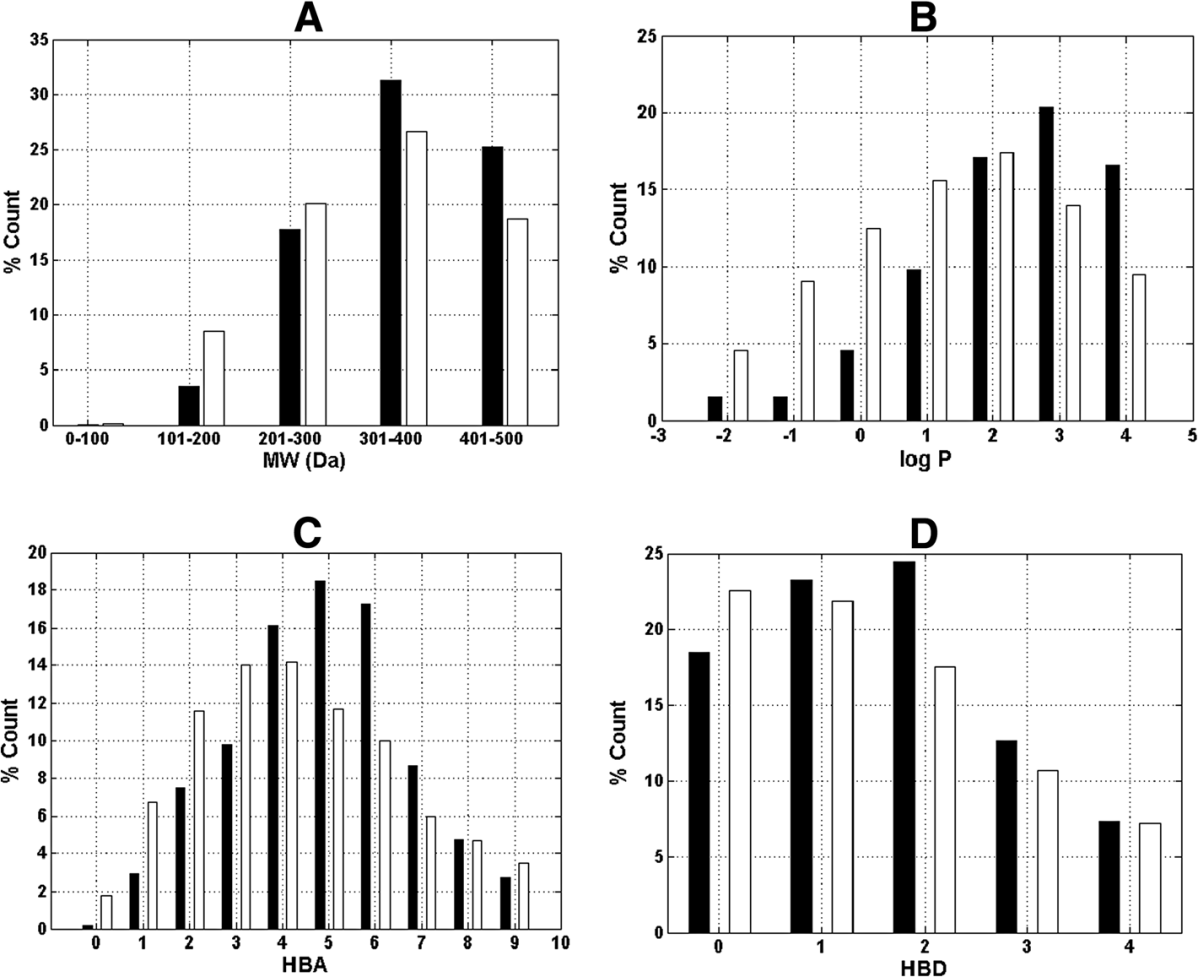
**Comparison of property distribution for the CamMedNP and DNP data sets.** (**A**) molar weight, (**B**) logarithm of octan-1-ol/water partition coefficient, (**C**) hydrogen bond acceptors and (**D**) hydrogen bond donors. DNP in white and CamMedNP in black. For subfigure **B**, the *x*-axis label is the lower limit of binned data, e.g. -2 is equivalent to −2 to −1.

A comparison of the distributions for the individual parameters for CamMedNP and the DNP is shown in Figure 5. In these histograms, we show only data that falls within the “Lipinski region of interest” (MW < 500, -2 < log *P* < 5, HBA < 10, and HBD < 5), and the values are expressed as a percentage count of their respective databases. In all cases the distributions of CamMedNP were enhanced for the Lipinski properties (peaks of the distributions moved to more “drug-like” properties) when compared to the DNP. The MW distribution (Figure 5A) peaks at 301–400 Da for both the DNP and the CamMedNP. The percentage of MW in the range 301–500 is significantly higher for CamMedNP. Below this range the percentages were reduced for the CamMedNP when compared to the DNP.

This improved profile for MW is exactly what is desirable for a more “drug-like” library, according to Lipinski’s criteria. The proportions of the two databases that satisfy Lipinski’s MW property (<500 Da) were 73% for DNP and 78% for CamMedNP. The distribution maxima for calculated log *P* (Figure 5B) were similar for both databases, with CamMedNP appearing between log *P* values of 3–4 and DNP giving a value between 2 and 3. A similar trend was observed for the MW distribution. This showed an enhancement of 11.2% for MW values between 301 and 500 Da of CamMedNP over the DNP and a corresponding 13.1% enhancement for log *P* values between 2 and 5 units. For HBA and HBD respectively (Figures 5C-D), CamMedNP showed improvements of 18.7% for 3 < HBA < 8 and 10.3 % for 0 < HBD < 4 over the DNP. The peak of the distribution for the HBA for the CamMedNP is at 5 acceptors (18.5%) with a significant increase in 6 or 7 acceptors when compared to the DNP (Figure 5C). Similarly, the peak of the distribution for the HBD for the CamMedNP is at 2 acceptors (24.5%) with a significant increase in 1 or 2 donors as compared to the DNP (Figure 5D). The overall summary of the four Lipinski parameters for the two databases, thus reveals that the CamMedNP library is more “drug-like” than the DNP. This is an indication that the chances of finding “lead-like” molecules with improved DMPK properties within a library such as CamMedNP are quite significant. The descriptors useful in ADMET prediction will also be included in the searchable version of the CamMedNP database.

### Diversity analysis

**Figure 6:**
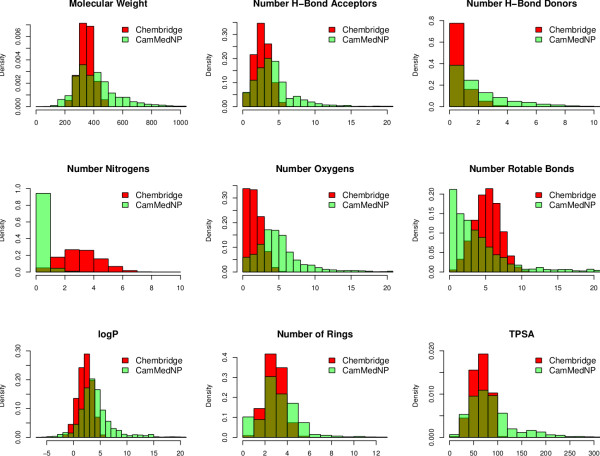
**A simple descriptor-based comparison of the CamMedNP database and the Chembridge Diversity database.** Comparison of typical physicochemical property distributions (MW, HBA, HBD, NN, NO, NRB, log *P*, NR and TPSA) in the CamMedNP (green) and ChemBridge Diverset (red) database. All histograms and scatterplots were generated with the R software [30].

**Figure 7:**
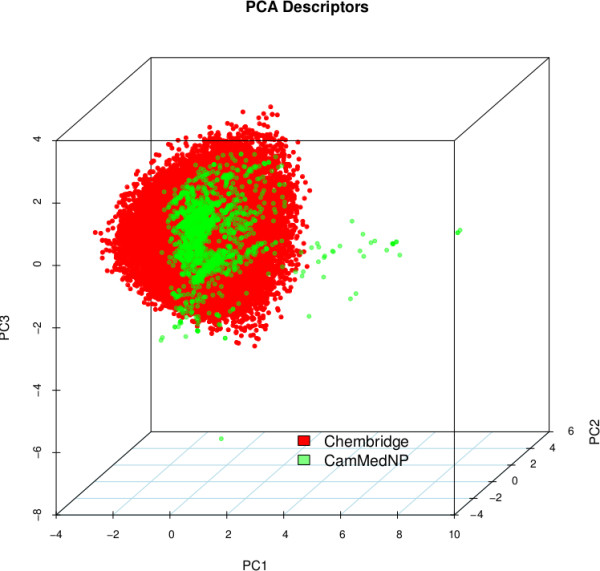
A principal component analysis (PCA) plot, showing the comparison of the chemical space defined by the NPs in CamMedNP (green) and the chemical space represented by NPs in the ChemBridge Diversity (red) databases.

In general, a diverse set of compounds should maximise the coverage of biological activity and minimise redundancy. The diversity of the CamMedNP database was analysed in comparison with a relatively large and diverse compound collection, namely the DIVERSet™ Database (48,651 compounds) from the ChemBridge Corporation [26]. A simple descriptor-based comparison of the CamMedNP database and the Chembridge Diversity database was carried out. The calculated descriptors include MW, HBA, HBD, log *P*, NR, NRB, NN, NO, NRB and TPSA (Figure 6). The regions of the histograms which are shown in dark green represent regions of intersection, while those of CamMedNP are shown in light green and those of ChemBridge in red. While the MW of the ChemBridge database is restricted to the range 200 ≤ MW ≤ 500 Da, that of CamMedNP stretches well beyond 1000 Da, an observations which could be explained by the complexity and large sizes of some of the NPs in CamMedNP. This remark could also explain the trend which is seen for the stretch in the distributions of log *P*, HBD, NO, NRB, NR, TPSA and HBA for CamMedNP, when compared with the ChemBridge dataset. On the contrary, the distribution of nitrogens for the ChemBridge database has a wider extent than that of CamMedNP. The overall results show that the CamMedNP database covers another physicochemical space than the ChemBridge Diversity database. The principal component analysis (PCA) scatter plot of the previously calculated physicochemical properties of the CamMedNP (green) and ChemBridge Diverset database (red) is shown in Figure 7. The first three principal components (PCs) explain 83% (CamMedNP) and 64% (ChemBridge) of the variance of the individual databases. The larger number of outliers in the case of the CamMedNP database indicates a wider sampling of the chemical space compared to the ChemBridge Diverset collection.

### Usefulness of the CamMedNP library

The usefulness of the CamMedNP database in lead generation has been exemplified with the docking and pharmacophore-based screening for potential inhibitors of a validated anti-malarial drug target in our laboratory, and the results will be published in a subsequent paper. CamMedNP is constantly being updated; meanwhile a computer program to facilitate the searching of this database is under development and will also be published subsequently. However, 3D structures of the compounds, as well as their physico-chemical properties that were used to evaluate “drug-likeness”, can be freely downloaded as a supplementary file accompanying this publication. In addition, information about compound sample availability can be obtained on request from the authors of this paper or from the pan-African Natural Products Library (p-ANAPL) project [31,32].

## Conclusions

Virtual screening workflows usually involve docking a compound library into the binding site of a target receptor and using scoring functions and binding free energy calculations to identify putative binders. The availability of 3D structures of the compounds to be used for docking is of utmost importance. Therefore the availability of such structures within CamMedNP, as well as their calculated physico-chemical properties and indicators of “drug-likeness” within this newly developed database will facilitate the drug discovery process from leads that have been identified from Cameroonian medicinal plants.

## Availability and requirements

3D structures of the compounds, as well as their physico-chemical properties that were used to evaluate “drug-likeness”, can be freely downloaded (for non commercial uses) as a supplementary file accompanying this publication (Additional file 2). Physical samples for testing are available at the various research labs in Cameroon in varying quantities. Questions regarding the available of compound samples could be addressed directly to the authors of this paper. Otherwise samples could be obtainable from the p-ANAPL consortium, which has a mandate to collect samples of NPs from the entire continent of Africa and make them available for biological screening. This network is being set up under the auspices of the Network for Analytical and Bioassay Services in Africa (NABSA) [31,32].

## Abbreviations

3D: Three dimensional; ADME/T: Absorption, distribution, metabolism, excretion, and toxicology; CADD: Computer-aided drug design; CamMedNP: Cameroonian Medicinal Plant and Natural Products Database; DMPK: Drug metabolism and pharmacokinetics; DNP: Dictionary of Natural Products; HBA: Hydrogen bond acceptors; HBD: Hydrogen bond donors; log P: logarithm of the octan-1-ol/water partition coefficient; MW: Molar weight; NABSA: Network for Analytical and Bioassay Services in Africa; NN: Number of nitrogens; NO: Number of oxygens; NP: Natural product; NR: Number of rings; NRB: Number of rotatable bonds; p-ANAPL: pan-African Natural Products Library; PCA: Principal component analysis; TPSA: Total polar surface area; VS: Virtual screening.

## Competing interests

The authors declare no conflicts of interest.

## Authors’ contributions

WS, LMM, FCN and SMNE conceived the idea. FNK, JAM, PAO, and JNH participated in the data collection and generation of 3D models. All authors contributed in the data analysis, the discussion of results and the conception of the paper. FNK wrote the first draft of the paper and all authors agreed on the final version before submission. This work is part of the PhD project of FNK.

## Authors’ information

WS and SMNE are professors of medicinal chemistry with an interest in CADD, while SMNE also focuses organic synthesis and on natural product leads from Cameroonian medicinal plants. LMM and JAM are natural product chemists actively involved in the isolation and characterization of secondary metabolites from Cameroonian medicinal plants. FCN is a biochemist/molecular biologists interested in docking and *in silico* screening. LLL holds a PhD in environmental chemistry and manages a Chemical and Bioactivity Information centre with a focus on developing databases for information from medicinal herbs in Africa. FNK is a PhD student working on CADD under the joint supervision of LCOO and EM, while PAO is an MSc student supervised by LMM, MS is a PhD student under the supervision of WS and JNH is a PhD student supervised by SMNE.

## Pre-publication history

The pre-publication history for this paper can be accessed here:

http://www.biomedcentral.com/1472-6882/13/88/prepub

## Supplementary Material

Additional file 1 Full list of consulted journals in constructing CamMedNP. Click here for file

Additional file 2 **3D structures of compounds currently included in CamMedNP.** This file is saved in .mdb format (which can be viewed using MOE) and could be converted into .sdf, .mol, or .mol2 (using the software MOE) or into .ldb format using the software LigandScout. Click here for file
