# Supplementary material for: CamMedNP: Building the Cameroonian 3D structural natural products database for virtual screening
Source: BMC Complement Altern Med. 2013 Apr 16;13:88. doi: 10.1186/1472-6882-13-88 (PMC3637470; doi:10.1186/1472-6882-13-88)
Supplement: Additional file 1 — Full list of consulted journals in constructing CamMedNP. [file 1472-6882-13-88-S1.doc]

# SUPPLEMENTARY MATERIAL

# Title: CamMedNP: Building the Cameroonian 3D Structural Natural Products Database for Virtual Screening

### Fidele Ntie-Kang^1,2§^, James Ajeck Mbah^3^, Luc Mbaze Meva’a^4^, Lydia Likowo Lifongo^3^, Joelle Ngo Hanna^3,4^, Fidelis Cho-Ngwa^5^, Pascal Amoa Onguéné^4^, Luc Calvin Owono Owono^2,6^, Eugene Megnassan^7^, Wolfgang Sippl^1^, Simon Mbua Ngale Efange^3^.

^1^Department of Pharmaceutical Sciences, Martin-Luther University of Halle-Wittenberg, Wolfgang-Langenbeck Str. 4, 06120, Halle (Saale), Germany

^2^CEPAMOQ, Faculty of Science, University of Douala, P.O. Box 8580, Douala, Cameroon

^3^Department of Chemistry, Faculty of Science, University of Buea, P. O. Box 63, Buea, Cameroon

^4^Department of Chemistry, Faculty of Science, University of Douala, P. O. Box 24157, Douala, Cameroon

^5^Department of Biochemistry and Molecular Biology, Faculty of Science, University of Buea, P. O. Box 63, Buea, Cameroon

^6^Department of Physics, Ecole Normale Supérieure, University of Yaoundé I, P.O. Box 47, Yaoundé, Cameroon

^7^Laboratory of Fundamental and Applied Physics, University of Abobo-Adjame, Abidjan 02 BP 801, Cote d’Ivoire

^§^Corresponding author

Email addresses: [ntiekfidele@gmail.com](mailto:ntiekfidele@gmail.com)

List of Journals Consulted in Constructing CamMedNP

| Journal type | List |
| --- | --- |
| International | *Acta Crystallographica*, *African Journal of Health Sciences*, *Asian Journal of Traditional Medicine*, *Biochemical Systematics and Ecology*, *Bioorganic and Medicinal Chemistry*, *Bioscience Biotechnology and Biochemistry*, *BMC Complementary and Alternative Medicine*, *BMC Research Notes*, *Boletín Latinoamericano y del Caribe de Plantas Medicinales y Aromáticas*, *Bulletin of the Chemical Society of Ethiopia*, *Carbohydrate Research*, *Chemistry and Biodiversity*, *Chemical and Pharmaceutical Bulletin*, *Fitoterapia*, *Helvetica Chimica Acta*, *Inflammopharmacology*, *Journal of Natural Products*, *Journal of Asian Natural Products Research*, *Journal of Ethnopharmacology*, *Journal of the American Oil Chemistry Society*, *Malaria Journal*, *Molecules*, *Natural Product Communications*, *Natural Product Letters*, *Natural Product Research*, *Natural Product Science*, *Pakistani Journal of Medical Science*, *Parasitology Research*, *Pharmacologia*, *Pharmacologyonline*, *Pharmazie*, *Phytochemistry*, *Phytochemistry Letters*, *Pharmaceutical Biology*, *Phytotherapy Research*, *Phytomedicine*, *Planta Medica*, *PLoS One*, *Pure and Applied Chemistry*, *Rasayan Journal of Chemistry*, *Records of Natural Products*, *Research Journal in Phytochemistry*, *South African Journal of Botany*, *Tetrahedron*, *Tetrahedron Letters*  and *Zeitschrift für Naturforschung*. |
| Cameroonian | *Journal of the Cameroonian Academy of Sciences*, *Cameroon Journal of Biosciences* and *Les Annales des la Faculté des Sciences des l’Université de Yaoundé I.* |
